# Supplementary material for: Upregulated hepatic lipogenesis from dietary sugars in response to low palmitate feeding supplies brain palmitate
Source: Nat Commun. 2024 Jan 17;15:490. doi: 10.1038/s41467-023-44388-4 (PMC10794264; doi:10.1038/s41467-023-44388-4)
Supplement: Supplementary file 1 — Supplementary Information [file 41467_2023_44388_MOESM1_ESM.pdf]

**Supplemental Material In:** Upregulated Hepatic Lipogenesis from Dietary Sugars in Response to Low Palmitate Feeding Supplies Brain Palmitate

**Authors:** Mackenzie E Smith[1], Chuck T Chen[1], Chiraag Gohel[2], Giulia Cisbani[1], Daniel K Chen[1], Kimia Rezaei[1], Andrew McCutcheon[1], Richard P. Bazinet*[1]

**Author Affiliations:** [1]Department of Nutritional Sciences, University of Toronto, 1 King's College Circle, Toronto, Ontario, Canada, M5S 1A8; [2]Department of Biostatistics and Bioinformatics, George Washington University, 950 New Hampshire Ave, NW, Washington, DC 20052, United States

***Corresponding Author Contact:** richard.bazinet@utoronto.ca

**Supplemental Figure 1: Male Pup Brain Palmitic Acid is Maintained in Individual Brain Phospholipid Fractions in Response to Diets.** Levels of palmitic acid (PAM) in all individual brain phospholipid (PL) fractions do not appear to be influenced by diet at postnatal day (P) 0 (**A**) and P10 (**B**). There was also not a significant effect of diet at P21 (**C**) and day 35 (**D**), only a significant effect of PL fraction at P21 ($p < 0.0001$) (**C**) and day 35 ($p < 0.0001$) (**D**). Data are biological replicates presented as mean ± SEM analyzed by two-way analysis of variance (ANOVA) (**C, D**). Because of limited sample size at P0 and P10, statistics were not conducted (**A, B**). n = 2, 3, 5 male mice per diet at P0 ethanolamine glycerophospholipids (PE), phosphatidylinositol (PI), phosphatidylserine (PS), sphingomyelin (SM) (**A**); n = 2, 3, 4 male mice per diet at P0 choline glycerophospholipids (PC) (**A**); n = 4, 3, 7 male mice per diet at P10 PC, PE, PI, PS, SM (**B**); n = 5, 6, 6 male mice per diet at P21 PC, PE, PS, SM (**C**); n = 5, 6, 5 male mice per diet at P21 PI (**C**); n = 5, 4, 4 male mice per diet at P35 PC, PE, PI, PS, SM (**D**). Individual data points represent concentration data in µmol/g (for which figure statistics correspond to) while the bars show relative percentage data. Source data are provided as a Source Data file. Med, medium.

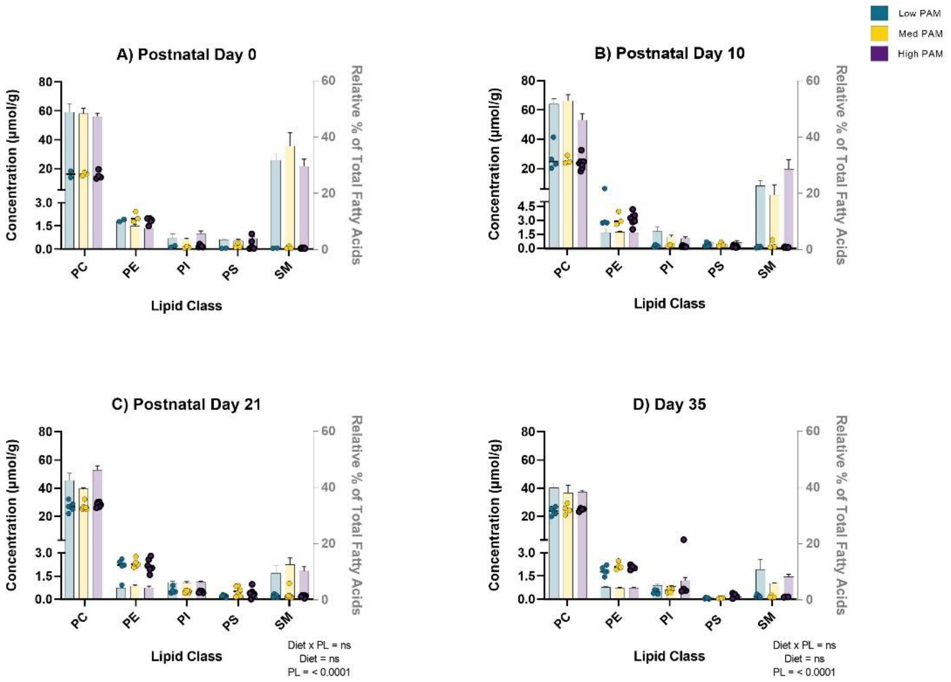

**Supplemental Figure 2: The Relative Percentage, but not the Concentration of Palmitic Acid is Reflective of Dietary Palmitic Acid Levels in Separated Male Liver Triacylglyceride and Cholesteryl Ester Fractions at Day 35.** There was a significant effect of diet in the relative percentage of palmitic acid (PAM) in separated male liver triacylglyceride (TAG) ($p < 0.0001$) (**A**) and cholesteryl ester (CE) ($p = 0.0135$) (**C**) fractions, however, not in liver phospholipid (PL) (**B**), monoacylglycerol (MAG) (**D**), diacylglycerol (DAG) (**E**) and free fatty acid (FFA) (**F**) fractions. The concentration of PAM in separated male liver neutral lipid fractions was variable overall and there was not a significant effect of diet (**A-F**). Data are biological replicates presented as mean ± SEM analyzed by ordinary one-way analysis of variance (ANOVA) (**C, E**) or Kruskal-Wallis test (**A, B, D, F**) for normally and non-normally distributed data, respectively. n = 5 male pups per diet (**A, B, C, D**), n = 5, 4, 5 mice per diet (**E**), n = 4, 5, 4 mice per diet (**F**). Individual data points represent concentration data in µmol/g (for which figure statistics correspond to) while the bars show relative percentage data. Source data are provided as a Source Data file. HP, high palmitic acid; LP, low palmitic acid; Med, medium; MP, medium palmitic acid.

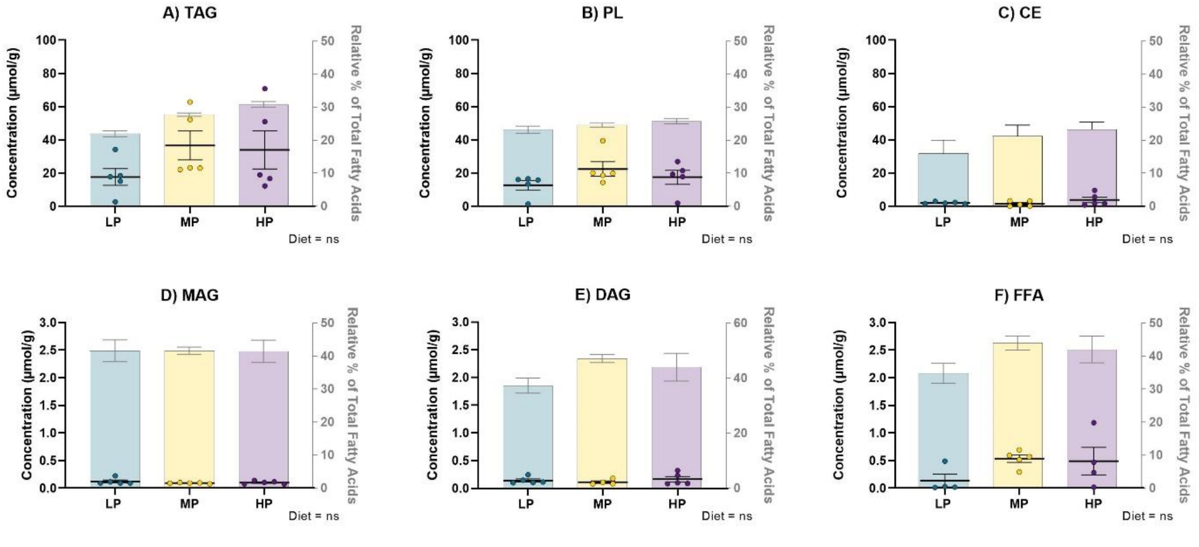

**Supplemental Figure 3: Female Pup Brain Palmitic Acid Levels are Maintained Compared to the Liver Primarily by Lipogenesis from Dietary Sugars Augmented in Mice fed Low Palmitic Acid.** While levels of palmitic acid (PAM) in the female liver appear impacted by time and diet (**B**), levels of PAM in the female brain do not appear to be impacted by diet, only time (**A**). Female pup tissue $\delta^{13}C$-PAM values are enriched overall and appear augmented in mice fed low compared to medium and high PAM in both the brain (**C**) and liver (**D**). Data are biological replicates presented as mean ± SEM. Because of limited sample size statistics were not conducted. n = 2, 2, 3; 3, 2, 2; 3, 3, 1; 2, 1, 2 female mice per diet per timepoint (**A-D**). Individual data points represent concentration data in µmol/g while the bars show relative percentage data (**A, B**). Source data are provided as a Source Data file. $\delta^{13}C$, $^{13}C/^{12}C$; Med, medium.

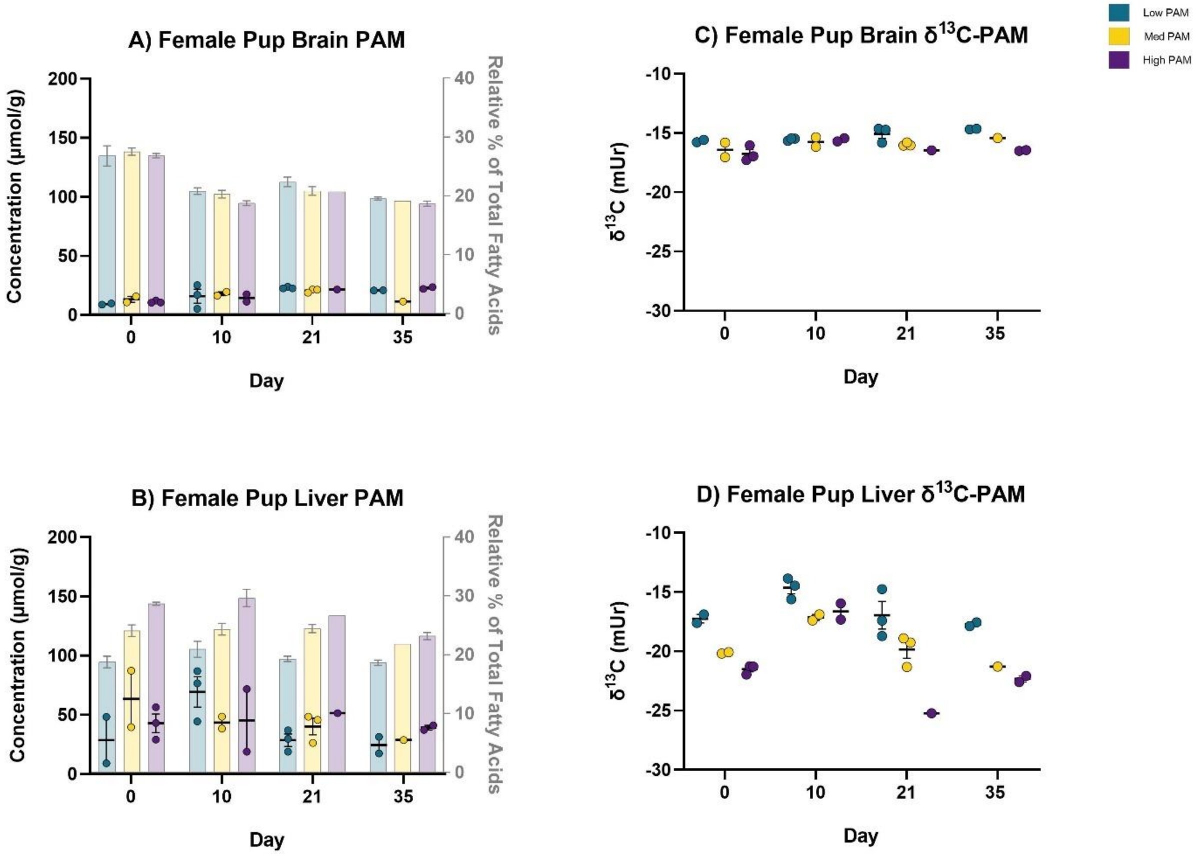

**Supplemental Figure 4: Levels of Lipogenic and Other Fatty Acids are Maintained in the Male Pup Brain in Response to Diet.** The concentration of lipogenic fatty acids including palmitoleic acid (POA) (**A**) and stearic acid (STA) (**B**) as well as essential fatty acids linoleic acid (LNA) (**D**) and arachidonic acid (ARA) (**E**) were maintained in the brain at all timepoints in response to diet. Interestingly, there was a significant interaction between diet and time in the concentration of brain oleic acid (OLA) (**C**) and docosahexaenoic acid (DHA) (**F**). Data are biological replicates presented as mean ± SEM analyzed by two-way analysis of variance (ANOVA) (**A-F**) and Tukey's multiple comparison test (**C, F**); for visualization purposes only means significantly different by diet at each timepoint are displayed; * < 0.03, **** < 0.0001. n = 8, 5, 8; 4, 5, 7; 7, 6, 7; 6, 5, 6 male pups per diet per timepoint (**A-F**). Individual data points represent concentration data in µmol/g (for which figure statistics correspond to) while the bars show relative percentage data. Source data are provided as a Source Data file. Med, medium.

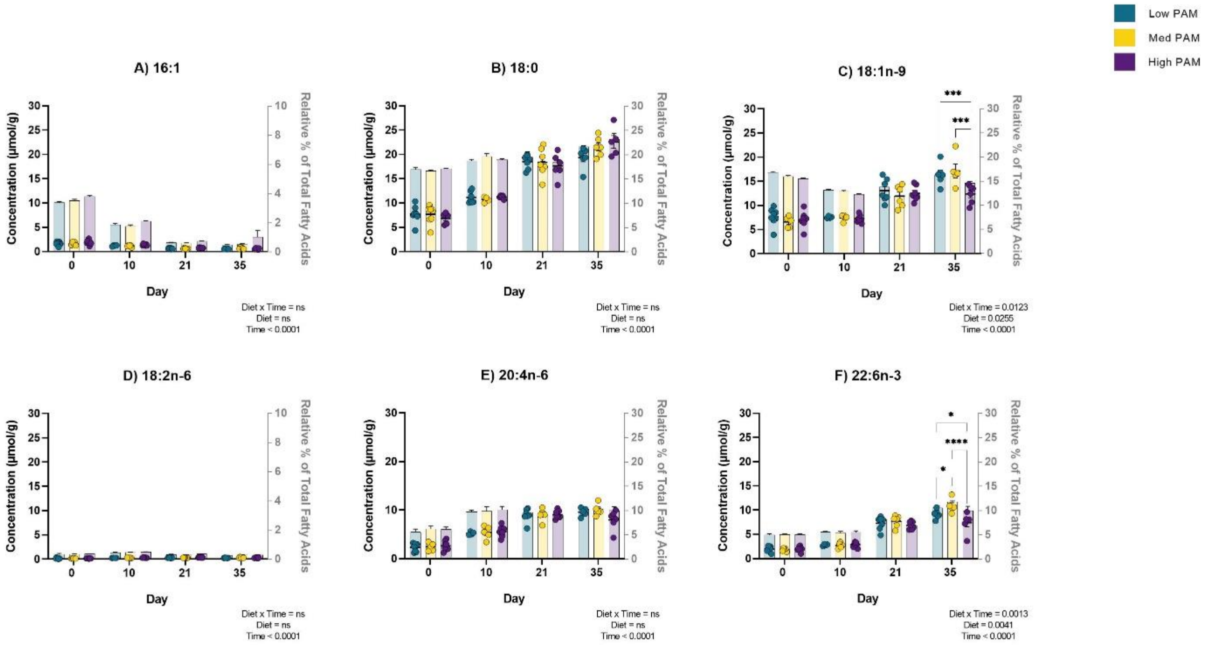

**Supplemental Figure 5: Levels of Lipogenic, but not Other Fatty Acids are Responsive to Diet in the Male Pup Liver.** There was a significant diet x time interaction in the concentration of lipogenic liver fatty acid palmitoleic acid (POA) (**A**), whereby mice fed low palmitic acid (PAM) had lower levels of liver POA than mice fed medium and high PAM at postnatal day (P) 0, but not P10, P21 and day 35. While liver stearic acid (STA) was not affected by diet (**B**), liver oleic acid (OLA) was significantly affected by diet whereby mice fed low PAM (high OLA) had higher levels of OLA than mice fed the medium, and high PAM diet (low OLA) at all timepoints (**C**). The concentration of liver essential fatty acid linoleic acid (LNA) (**D**), but not arachidonic acid (ARA) (**E**) nor docosahexaenoic acid (DHA) (**F**) was affected by diet. Data are biological replicates presented as mean ± SEM analyzed by two-way analysis of variance (ANOVA) (**A-F**) and Tukey's multiple comparison test (**A**); for visualization purposes only means significantly different by diet at each timepoint are displayed; *** < 0.0002, **** < 0.0001; n = 8, 5, 8; 4, 5, 7; 7, 6, 7; 6, 5, 6 male mice per diet per timepoint (**A-F**). Individual data points represent concentration data in µmol/g (for which figure statistics correspond to) while the bars show relative percentage data. Source data are provided as a Source Data file. Med, medium.

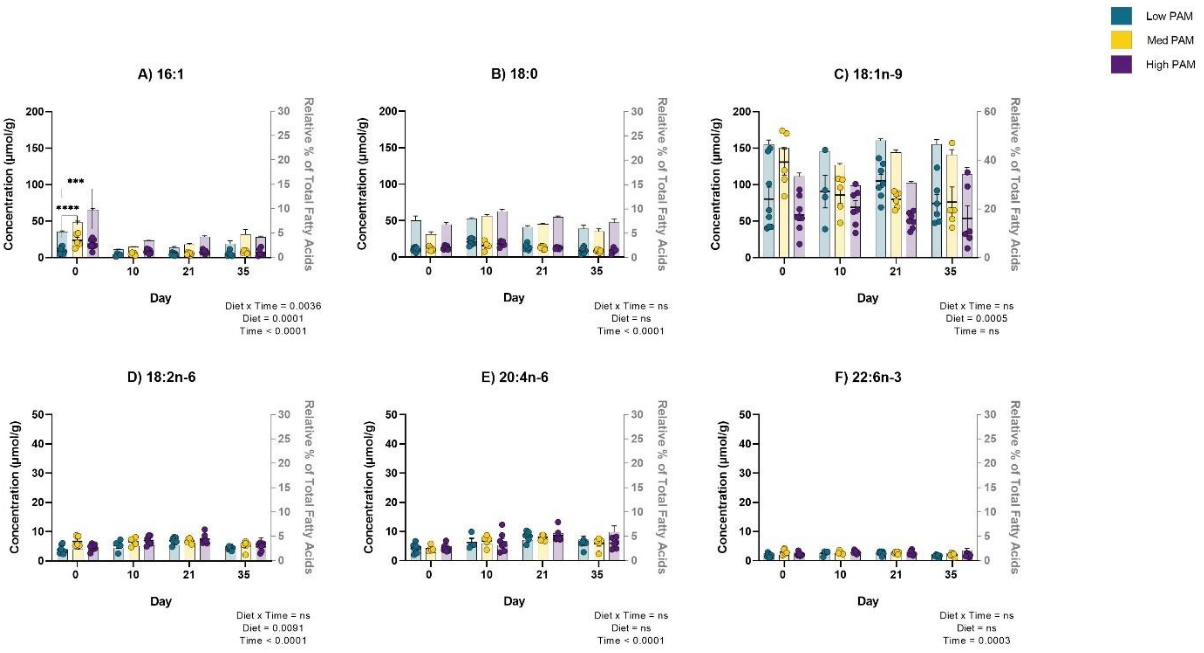

**Supplemental Figure 6: Male Liver δ$^{13}$C-Palmitic Acid in Separated Triacylglyceride, but not Other Separated Liver Fractions Responsive to Diet at Day 35.** There was a significant dose-response increase in δ$^{13}$C-palmitic acid (PAM) in response to diet in separated male liver triacylglyceride (TAG) (**A**) whereby mice fed low PAM had an increased δ$^{13}$C-PAM value compared to mice fed medium and high PAM. However, there was not a significant dose-response increase in δ$^{13}$C-PAM in response to diet in male separated liver phospholipid (PL) (**B**), cholesteryl ester (CE) (**C**), monoacylglycerol (MAG) (**D**), diacylglycerol (DAG) (**E**) and free fatty acid (FFA) (**F**) fractions. Data are biological replicates presented as mean ± SEM analyzed by ordinary one-way analysis of variance (ANOVA) (**A-F**). n = 5 male mice per diet (**A-D**), n = 3, 5, 5 male mice per diet (**E**), n = 4, 5, 5 male mice per diet (**F**). Source data are provided as a Source Data file. δ$^{13}$C, $^{13}C/^{12}C$; HP, high palmitic acid; LP, low palmitic acid; Med, medium; MP, medium palmitic acid.

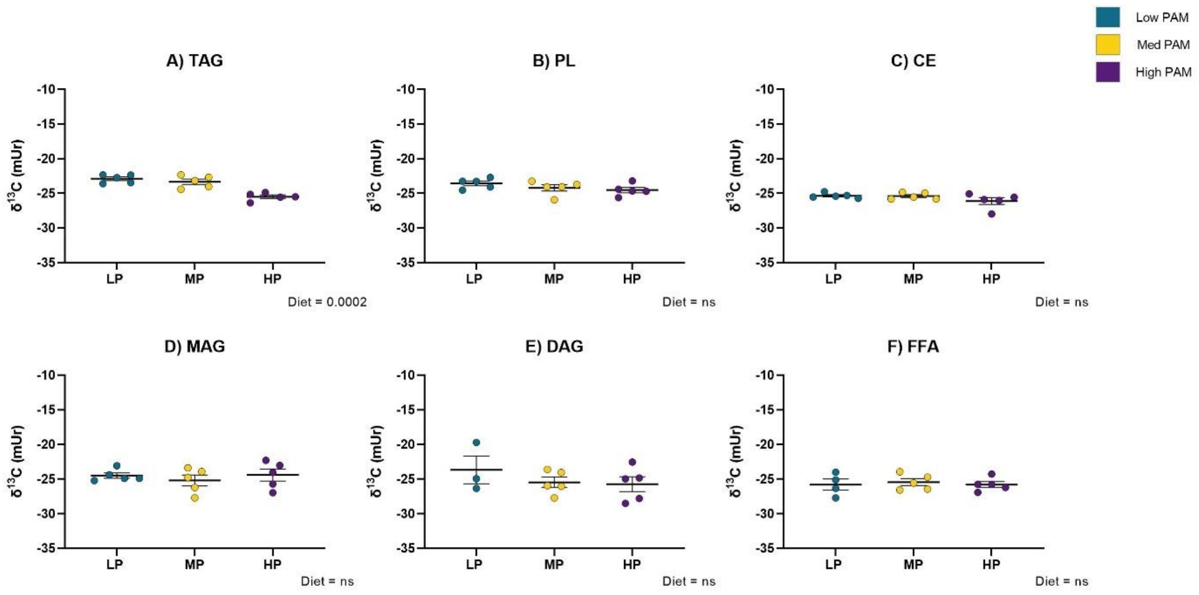

**Supplemental Figure 7: Lipogenic, but not Other Brain Fatty Acid δ$^{13}$C Values Impacted by Diet in Male Pups.** Male brain δ$^{13}$C-palmitoleic acid (POA) is enriched overall and is significantly augmented in mice fed low palmitic acid (PAM) compared to medium and high PAM (**A**). Male brain δ$^{13}$C-stearic acid (STA) and δ$^{13}$C-oleic acid (OLA) are enriched overall, whereby the δ$^{13}$C value depended on time (**B**, **C**). Compared to δ$^{13}$C values of lipogenic brain fatty acids (**A**, **B**, **C**), δ$^{13}$C values of essential brain fatty acids; linoleic acid (LNA) (**D**) and arachidonic acid (ARA) (**E**) are more deplete in δ$^{13}$C and are only significantly affected by time and not diet. Similarly, docosahexaenoic acid (DHA) is more deplete in δ$^{13}$C and is significantly affected by time and not diet (**F**). Data are biological replicates presented as mean ± SEM analyzed by two-way analysis of variance (ANOVA) (**A-F**) and Tukeys multiple comparison test (**B, C**); for visualization purposes only means significantly different by diet at each timepoint are displayed; * < 0.03, ** < 0.002, *** < 0.0002, **** < 0.0001. n = 8, 5, 8; 4, 5, 7; 6, 6, 7; 6, 5, 6 male mice per diet per timepoint (**A-F**). Source data are provided as a Source Data file. δ$^{13}$C, $^{13}$C/$^{12}$C; Med, medium.

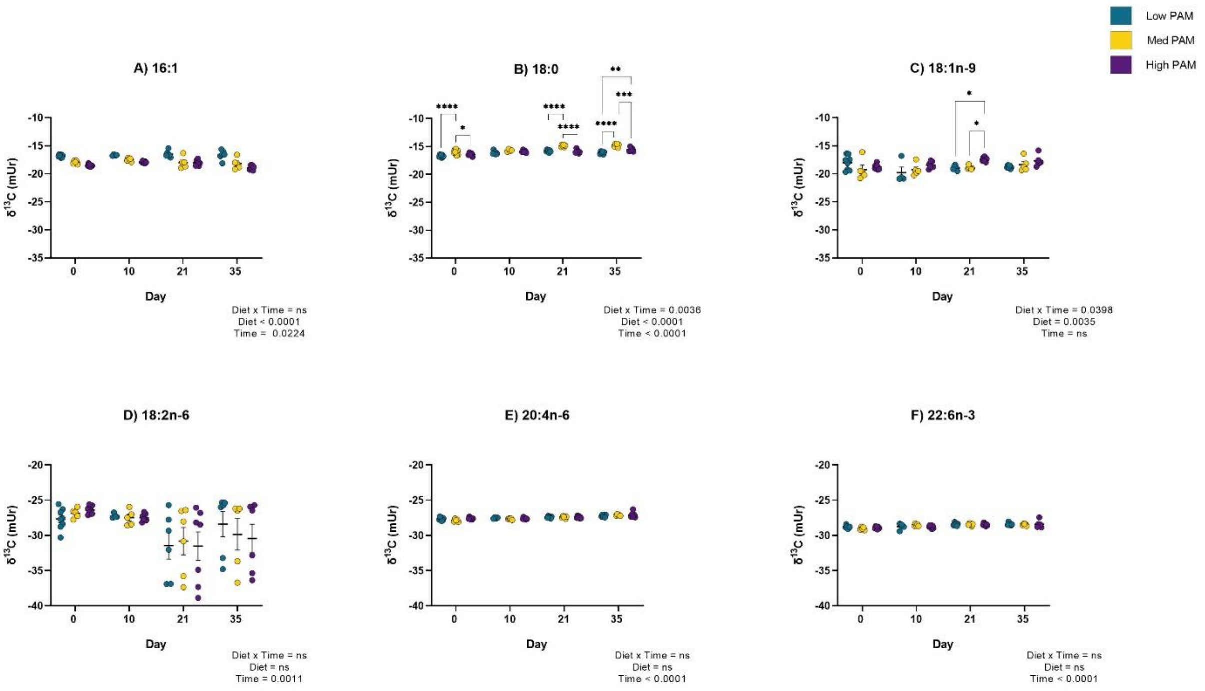

**Supplemental Figure 8: Lipogenic, but not Other Liver Fatty Acid δ$^{13}$C Values Impacted by Diet in Male Pups.** Male liver δ$^{13}$C-palmitoleic acid (POA) is enriched overall and is significantly augmented in mice fed low PAM compared to medium and high palmitic acid (PAM) (**A**), while male liver δ$^{13}$C-oleic acid (OLA) depends on time but is enriched overall and appears augmented in mice fed medium and high PAM compared to low PAM (**C**). Male liver δ$^{13}$C-stearic acid (STA) is also enriched overall, whereby the δ$^{13}$C value depends on time but appears augmented in mice fed low PAM compared to medium and high PAM (**B**). Compared to δ$^{13}$C values of lipogenic liver fatty acids (**A**, **B**, **C**), δ$^{13}$C values of essential liver fatty acids; linoleic acid (LNA) (**D**) and arachidonic acid (ARA) (**E**) are more deplete in δ$^{13}$C and only significantly affected by time, not diet. Similarly, docosahexaenoic acid (DHA), is more deplete in δ$^{13}$C and significantly affected by time and not diet (**F**). Data are biological replicates presented as mean ± SEM analyzed by two-way analysis of variance (ANOVA) (**A-F**) and Tukeys multiple comparison test (**B**); for visualization purposes only means significantly different by diet at each timepoint are displayed; ** < 0.002, *** < 0.0002, **** < 0.0001. n = 8, 6, 8; 4, 5, 7; 7, 6, 7; 6, 5, 6 male mice per diet per timepoint (**A-B, D-E**), n = 6, 4, 8; 4, 5, 7; 7, 6, 7; 6, 5, 6 (**C**); n = 8, 5, 8; 3, 5, 6; 7, 6, 7; 6, 5, 6 (**F**). Source data are provided as a Source Data file. δ$^{13}$C, $^{13}$C/$^{12}$C; Med, medium.

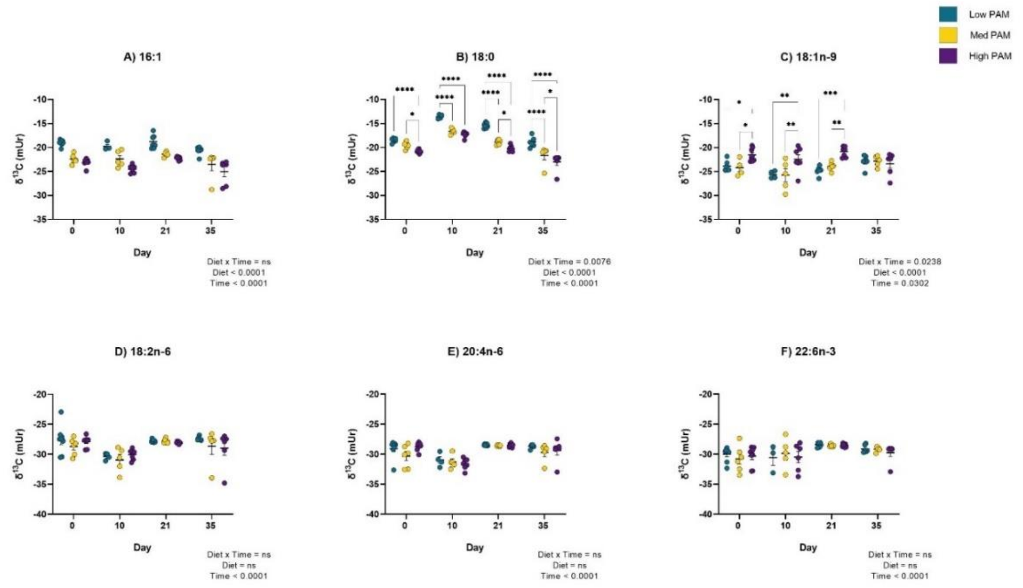

**Supplemental Figure 9: Maternal Behaviour and Pup Sensorimotor Development are Not Impacted by Diets.** There were no differences by diet in average pup litter scores for either the geotaxis test (**A**) nor the righting reflex test (**B**), however, pup scores for both tests improved with time (p < 0.0001 for both). Similarly, there were no differences by diet in dam nest scores between gestational days 15 to 18 (**D**). Data are biological replicates presented as mean ± SEM analyzed by two-way analysis of variance (ANOVA) (**A, B**) and ordinary one-way ANOVA (**D**). n = 8, 7, 8 average litter scores per diet (**A, B**); n = 6, 5, 6 dams per diet (**D**). One dam was excluded from the maternal next test in the MP group due to late impregnation. Source data are provided as a Source Data file. HP, high palmitic acid; LP, low palmitic acid; Med, medium; MP, medium palmitic acid; PAM, palmitic acid.

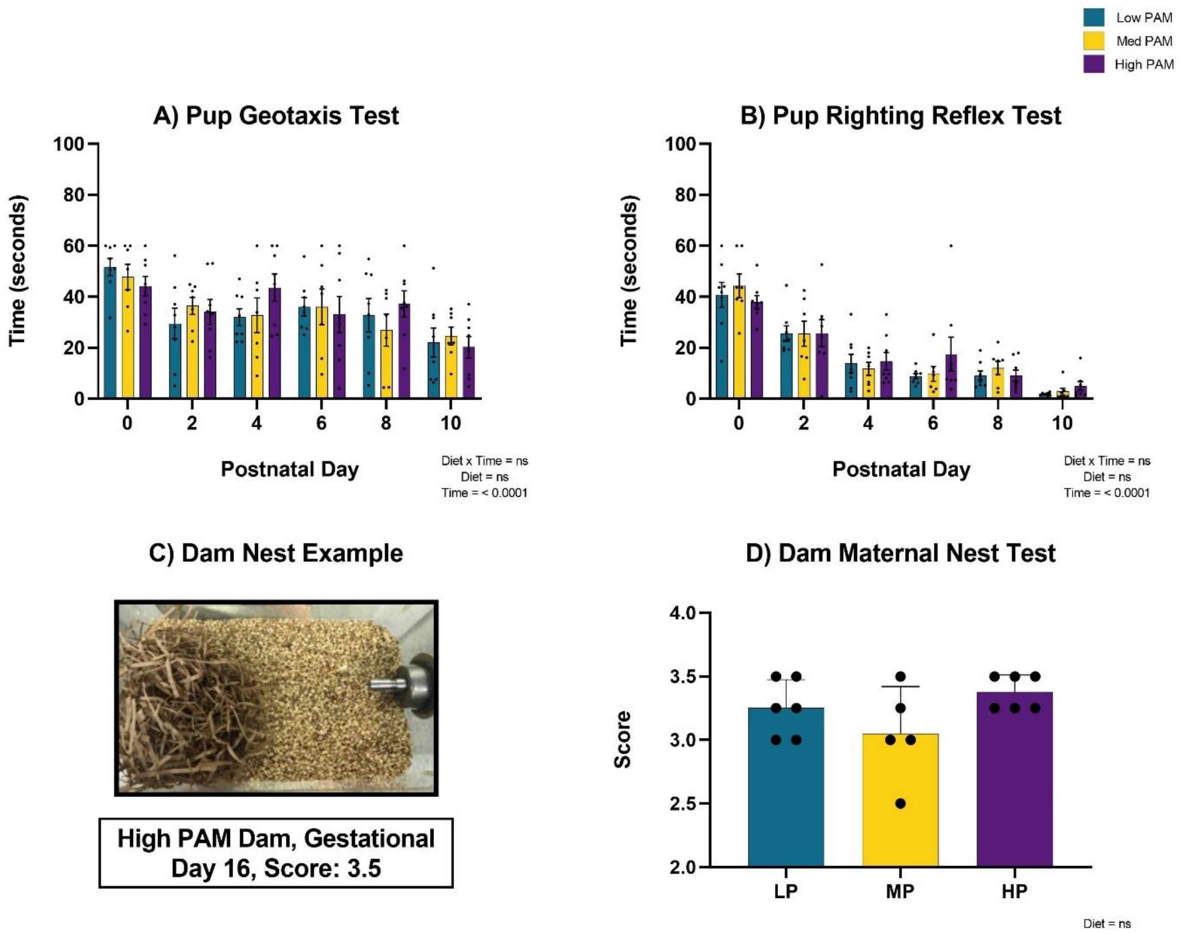

**Supplemental Figure 10: Heatmaps for Male Pup Liver and Brain Weighted Gene Co-Expression Network Analysis at Day 35.** Modules of highly correlated genes were explored by weighted gene co-expression network analysis (WGCNA) on the top 1000 most variable genes in the liver (**A**) and brain (**B**). n = 5 male mice per diet (biological replicates). Soft threshold was set to 6 and a minimum module size of 20 was used.

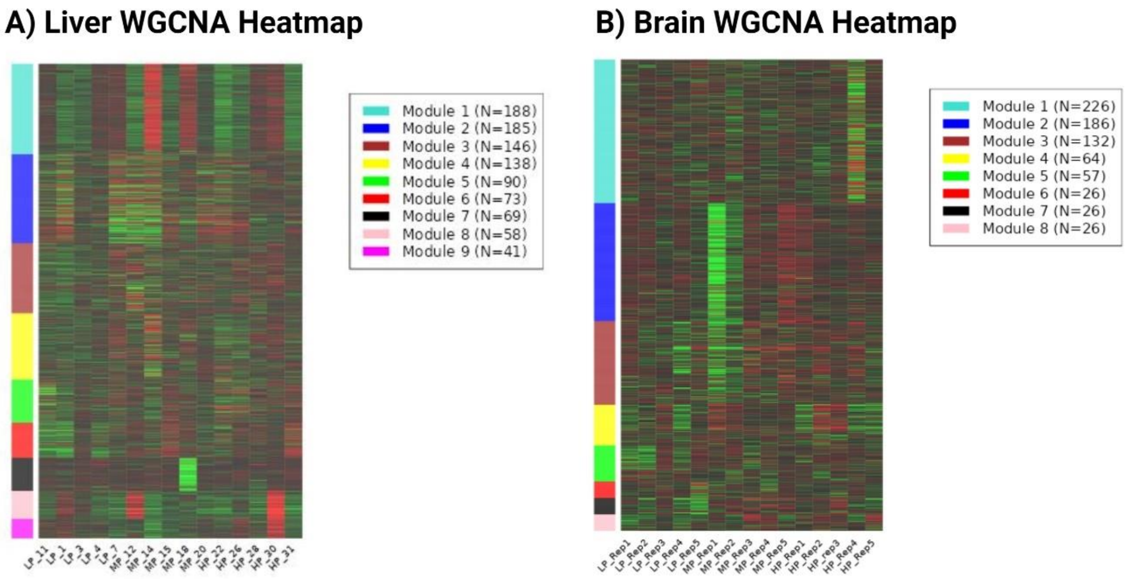

**Supplemental Figure 11: Male Pup Brain Gene Dendrogram and Module Colours at Day 35.** A network of 743 genes was divided into 8 modules by brain weighted gene co-expression network analysis (WGCNA). n = 5 male mice per diet (biological replicates). Soft threshold was set to 6 and a minimum module size of 20 was used.

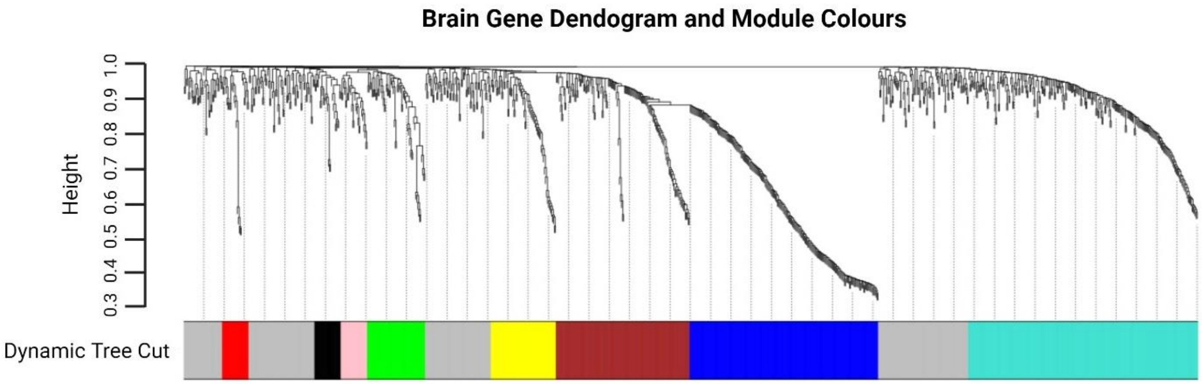

**Supplemental Figure 12: Dam Milk Palmitic Acid Levels are Reflective of Dietary Palmitic Acid Levels Pre-Weaning.** Levels of palmitic acid (PAM) in pup stomach content containing dam milk reflected dietary levels of PAM at postnatal day (P) 0 and P10. Data are biological replicates presented as mean ± SEM. Because sample size relied on a fed state, it was limited at P10 (n = 2 in low PAM group), therefore, statistics were not conducted. n = 4, 4, 7 male mice per diet at P0; n = 2, 5, 5 male mice per diet at P10. Individual data points represent concentration data in µmol/g while the bars show relative percentage data. Source data are provided as a Source Data file. Med, medium; PAM, palmitic acid.

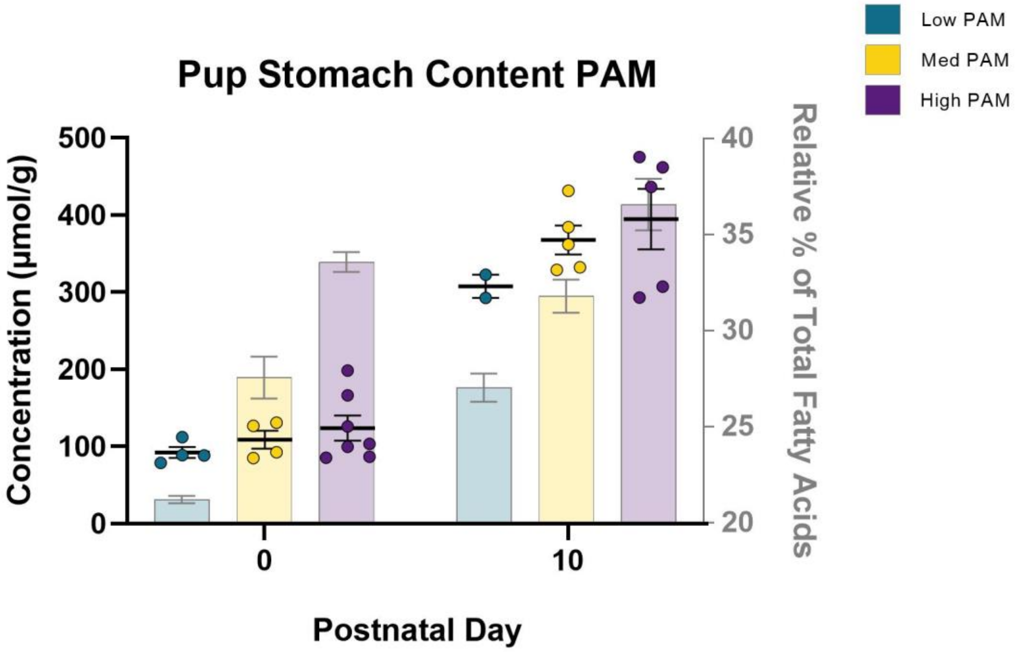

**Supplemental Figure 13: Litter Data.** There were no significant differences between diet groups in terms of total pups born per litter (**A**), number of male pups per litter (**B**) or number of female pups per litter (**C**). However, dams consistently had a higher number of male offspring compared to female offspring within their litters ($p < 0.0001$) (**D**), irrespective of diet. Data are biological replicates presented as mean ± SEM analyzed by ordinary one-way analysis of variance (ANOVA) (**A, B**) or Kruskal-Wallis test (**C**) for normally and non-normally distributed data, respectively, as well as two-way ANOVA (**D**). n = 11, 10, 11 litters per diet (**A**); n = 10, 9, 10 litters per diet (**B**); n = 10, 8, 9 litters per diet (**C**); n = 10, 10; 9, 8; 10, 9 litters per sex per diet (**D**). Litters that involved dam infanticide (n = 2) were excluded from this analysis. Source data are provided as a Source Data file. HP, high palmitic acid; LP, low palmitic acid; Med, medium; MP, medium palmitic acid; PAM, palmitic acid.

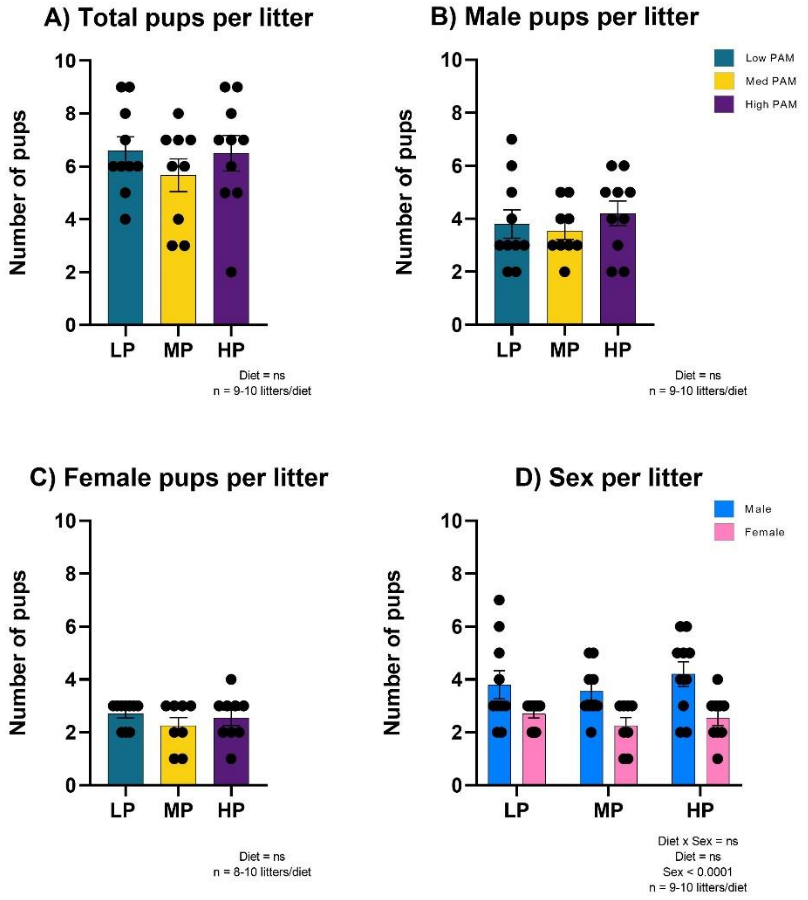

**Supplemental Figure 14: Studying Carbon Isotope Ratios at the Natural Abundance Level: A Summary of Tissue Fatty Acids and Their Origin.** A detailed overview of the three sources capable of maintaining the brain palmitic acid (PAM) pool; PAM sourced directly from the diet (**A**), PAM synthesized *via* lipogenesis from dietary sugars in the liver (**B**), and PAM synthesized *via* lipogenesis from dietary sugars locally in the brain (**C**). Importantly, tissue PAM of dietary origin is deplete in $\delta^{13}C$ (shown in blue), while PAM synthesized endogenously via de novo lipogenesis from dietary sugars is enriched in $\delta^{13}C$ (shown in red). ACC, acetyl-coA carboxylase; ACLY, ATP citrate lyase; DNL, de novo lipogenesis; FAS; fatty acid synthase; TCA, the citric acid cycle.  Created with BioRender.com.

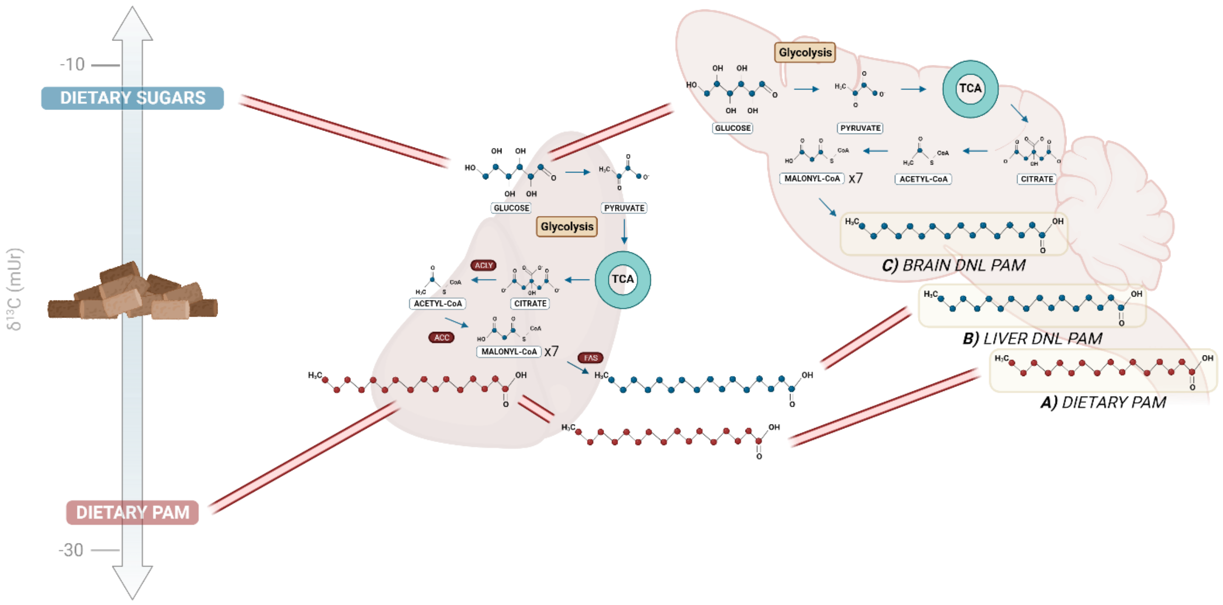

**Supplemental Table 1:** Tukey's Multiple Comparisons Test: Male Pup Mouse Brain $\delta^{13}$C-Palmitic Acid

| Tukey's multiple comparisons test | Predicted (LS) mean diff. | 95.00% CI of diff. | Below threshold? | Summary | Adjusted P Value |
|---|---|---|---|---|---|
| | | **Brain $\delta^{13}$C-PAM** | | | |
| | | | | | |
| 0 :Low PAM vs. 0 :Medium PAM | 0.6508 | 0.09889 to 1.203 | Yes | ** | 0.0085 |
| 0 :Low PAM vs. 0 :High PAM | 0.7900 | 0.3060 to 1.274 | Yes | **** | <0.0001 |
| 0 :Low PAM vs. 10:Low PAM | -0.1813 | -0.7740 to 0.4115 | No | ns | 0.9961 |
| 0 :Low PAM vs. 10:Medium PAM | 0.2387 | -0.3131 to 0.7906 | No | ns | 0.9434 |
| 0 :Low PAM vs. 10:High PAM | 0.4287 | -0.07225 to 0.9298 | No | ns | 0.1644 |
| 0 :Low PAM vs. 21:Low PAM | -0.6798 | -1.181 to -0.1788 | Yes | ** | 0.0012 |
| 0 :Low PAM vs. 21:Medium PAM | 0.3404 | -0.1824 to 0.8632 | No | ns | 0.5482 |
| 0 :Low PAM vs. 21:High PAM | 0.4559 | -0.04511 to 0.9569 | No | ns | 0.1082 |
| 0 :Low PAM vs. 35 :Low PAM | -0.6646 | -1.187 to -0.1418 | Yes | ** | 0.0031 |
| 0 :Low PAM vs. 35 :Medium PAM | 0.2948 | -0.2571 to 0.8466 | No | ns | 0.8043 |
| 0 :Low PAM vs. 35 :High PAM | 1.000 | 0.4776 to 1.523 | Yes | **** | <0.0001 |
| 0 :Medium PAM vs. 0 :High PAM | 0.1393 | -0.4126 to 0.6911 | No | ns | 0.9993 |
| 0 :Medium PAM vs. 10:Low PAM | -0.8320 | -1.481 to -0.1826 | Yes | ** | 0.0028 |
| 0 :Medium PAM vs. 10:Medium PAM | -0.4120 | -1.024 to 0.2002 | No | ns | 0.4976 |
| 0 :Medium PAM vs. 10:High PAM | -0.2220 | -0.7888 to 0.3448 | No | ns | 0.9717 |
| 0 :Medium PAM vs. 21:Low PAM | -1.331 | -1.897 to -0.7638 | Yes | **** | <0.0001 |
| 0 :Medium PAM vs. 21:Medium PAM | -0.3103 | -0.8965 to 0.2758 | No | ns | 0.8129 |
| 0 :Medium PAM vs. 21:High PAM | -0.1949 | -0.7617 to 0.3720 | No | ns | 0.9896 |
| 0 :Medium PAM vs. 35 :Low PAM | -1.315 | -1.902 to -0.7292 | Yes | **** | <0.0001 |
| 0 :Medium PAM vs. 35 :Medium PAM | -0.3560 | -0.9682 to 0.2562 | No | ns | 0.7080 |
| 0 :Medium PAM vs. 35 :High PAM | 0.3497 | -0.2365 to 0.9358 | No | ns | 0.6747 |
| 0 :High PAM vs. 10:Low PAM | -0.9713 | -1.564 to -0.3785 | Yes | **** | <0.0001 |
| 0 :High PAM vs. 10:Medium PAM | -0.5513 | -1.103 to 0.0006092 | No | ns | 0.0505 |
| 0 :High PAM vs. 10:High PAM | -0.3613 | -0.8623 to 0.1398 | No | ns | 0.3908 |
| 0 :High PAM vs. 21:Low PAM | -1.470 | -1.971 to -0.9688 | Yes | **** | <0.0001 |
| 0 :High PAM vs. 21:Medium PAM | -0.4496 | -0.9724 to 0.07321 | No | ns | 0.1594 |

| | | | | | |
|---|---|---|---|---|---|
| 0 :High PAM vs. 21:High PAM | -0.3341 | -0.8351 to 0.1669 | No | ns | 0.5116 |
| 0 :High PAM vs. 35 :Low PAM | -1.455 | -1.977 to -0.9318 | Yes | **** | <0.0001 |
| 0 :High PAM vs. 35 :Medium PAM | -0.4953 | -1.047 to 0.05661 | No | ns | 0.1196 |
| 0 :High PAM vs. 35 :High PAM | 0.2104 | -0.3124 to 0.7332 | No | ns | 0.9656 |
| 10:Low PAM vs. 10:Medium PAM | 0.4200 | -0.2294 to 1.069 | No | ns | 0.5584 |
| 10:Low PAM vs. 10:High PAM | 0.6100 | 0.003258 to 1.217 | Yes | * | 0.0476 |
| 10:Low PAM vs. 21:Low PAM | -0.4986 | -1.105 to 0.1082 | No | ns | 0.2097 |
| 10:Low PAM vs. 21:Medium PAM | 0.5217 | -0.1032 to 1.147 | No | ns | 0.1913 |
| 10:Low PAM vs. 21:High PAM | 0.6371 | 0.03040 to 1.244 | Yes | * | 0.0314 |
| 10:Low PAM vs. 35 :Low PAM | -0.4833 | -1.108 to 0.1415 | No | ns | 0.2879 |
| 10:Low PAM vs. 35 :Medium PAM | 0.4760 | -0.1734 to 1.125 | No | ns | 0.3659 |
| 10:Low PAM vs. 35 :High PAM | 1.182 | 0.5568 to 1.807 | Yes | **** | <0.0001 |
| 10:Medium PAM vs. 10:High PAM | 0.1900 | -0.3768 to 0.7568 | No | ns | 0.9915 |
| 10:Medium PAM vs. 21:Low PAM | -0.9186 | -1.485 to -0.3518 | Yes | **** | <0.0001 |
| 10:Medium PAM vs. 21:Medium PAM | 0.1017 | -0.4845 to 0.6878 | No | ns | >0.9999 |
| 10:Medium PAM vs. 21:High PAM | 0.2171 | -0.3497 to 0.7840 | No | ns | 0.9760 |
| 10:Medium PAM vs. 35 :Low PAM | -0.9033 | -1.490 to -0.3172 | Yes | *** | 0.0001 |
| 10:Medium PAM vs. 35 :Medium PAM | 0.05600 | -0.5562 to 0.6682 | No | ns | >0.9999 |
| 10:Medium PAM vs. 35 :High PAM | 0.7617 | 0.1755 to 1.348 | Yes | ** | 0.0023 |
| 10:High PAM vs. 21:Low PAM | -1.109 | -1.626 to -0.5911 | Yes | **** | <0.0001 |
| 10:High PAM vs. 21:Medium PAM | -0.08833 | -0.6269 to 0.4502 | No | ns | >0.9999 |
| 10:High PAM vs. 21:High PAM | 0.02714 | -0.4903 to 0.5446 | No | ns | >0.9999 |
| 10:High PAM vs. 35 :Low PAM | -1.093 | -1.632 to -0.5548 | Yes | **** | <0.0001 |
| 10:High PAM vs. 35 :Medium PAM | -0.1340 | -0.7008 to 0.4328 | No | ns | 0.9996 |
| 10:High PAM vs. 35 :High PAM | 0.5717 | 0.03311 to 1.110 | Yes | * | 0.0281 |
| 21:Low PAM vs. 21:Medium PAM | 1.020 | 0.4817 to 1.559 | Yes | **** | <0.0001 |
| 21:Low PAM vs. 21:High PAM | 1.136 | 0.6183 to 1.653 | Yes | **** | <0.0001 |
| 21:Low PAM vs. 35 :Low PAM | 0.01524 | -0.5233 to 0.5538 | No | ns | >0.9999 |
| 21:Low PAM vs. 35 :Medium PAM | 0.9746 | 0.4078 to 1.541 | Yes | **** | <0.0001 |
| 21:Low PAM vs. 35 :High PAM | 1.680 | 1.142 to 2.219 | Yes | **** | <0.0001 |
| 21:Medium PAM vs. 21:High PAM | 0.1155 | -0.4231 to 0.6540 | No | ns | 0.9999 |
| 21:Medium PAM vs. 35 :Low PAM | -1.005 | -1.564 to -0.4461 | Yes | **** | <0.0001 |
| 21:Medium PAM vs. 35 :Medium PAM | -0.04567 | -0.6318 to 0.5405 | No | ns | >0.9999 |

| | | | | | |
|---|---|---|---|---|---|
| 21:Medium PAM vs. 35 :High PAM | 0.6600 | 0.1011 to 1.219 | Yes | ** | 0.0084 |
| 21:High PAM vs. 35 :Low PAM | -1.120 | -1.659 to -0.5819 | Yes | **** | <0.0001 |
| 21:High PAM vs. 35 :Medium PAM | -0.1611 | -0.7280 to 0.4057 | No | ns | 0.9979 |
| 21:High PAM vs. 35 :High PAM | 0.5445 | 0.005964 to 1.083 | Yes | * | 0.0452 |
| 35 :Low PAM vs. 35 :Medium PAM | 0.9593 | 0.3732 to 1.546 | Yes | **** | <0.0001 |
| 35 :Low PAM vs. 35 :High PAM | 1.665 | 1.106 to 2.224 | Yes | **** | <0.0001 |
| 35 :Medium PAM vs. 35 :High PAM | 0.7057 | 0.1195 to 1.292 | Yes | ** | 0.0066 |

n = 8, 5, 8; 4, 5, 7; 7, 6, 7; 6, 5, 6 male pups per diet per timepoint (biological replicates). 0, postnatal day 0; 10, postnatal day 10; 21, postnatal day 21; 35, day 35; $\delta^{13}C$, $^{13}C/^{12}C$; CI, confidence interval; diff, difference; LS, least squares; PAM, palmitic acid.

**Supplemental Table 2.** Top Differentially Expressed Genes in Male Liver by Differential Gene Analysis at Day 35

| Gene Name | Gene Type | Log2 Fold Change | P-Value | Adjusted P-Value |
|---|---|---|---|---|
| **Adjusted P-Value < 0.05; Log2 Fold Change > 1.2** | | | | |
| **High PAM Diet vs. Low PAM Diet** | | | | |
| Cyp7a1 | Protein coding | 3.9724 | 5.9E-10 | 0.0000 |
| Hes1 | Protein coding | -1.4532 | 4.7E-06 | 0.0344 |
| Synm | Protein coding | 1.6028 | 1.2E-05 | 0.0344 |
| Arrdc3 | Protein coding | -1.7612 | 1.3E-05 | 0.0344 |
| Jaml | Protein coding | 2.5400 | 1.5E-05 | 0.0352 |
| Gm8941 | Processed pseudogene | 1.3736 | 2E-05 | 0.0369 |
| **Medium PAM Diet vs. Low PAM Diet** | | | | |
| BC049987 | lncRNA | 1.8426 | 3.24E-07 | 0.0025 |
| Mroh6 | Protein coding | 2.2508 | 3.3E-06 | 0.0102 |
| Spr-ps1 | Transcribed unprocessed pseudogene | -2.0684 | 8.62E-06 | 0.0147 |
| Agap2 | Protein coding | 1.2878 | 9.06E-06 | 0.0147 |
| Mfsd2a | Protein coding | 1.2067 | 1.72E-05 | 0.0187 |
| Jun | Protein coding | 1.3876 | 2.43E-05 | 0.0193 |
| P4ha1 | Protein coding | 1.2183 | 2.49E-05 | 0.0193 |
| Esrrb | Protein coding | 2.0499 | 2.5E-05 | 0.0193 |
| Tat | Protein coding | 1.3202 | 2.85E-05 | 0.0199 |
| Pla2g4f | Protein coding | 3.6640 | 2.88E-05 | 0.0199 |
| Gm3953 | Processed pseudogene | 2.5932 | 9.95E-05 | 0.0496 |
| **Adjusted P-Value < 0.1; Log2 Fold Change > 1.2** | | | | |
| **High PAM Diet vs. Low PAM Diet** | | | | |
| Cyp7a1 | Protein coding | 3.9724 | 5.91E-10 | 9.43E-06 |
| Hes1 | Protein coding | -1.4532 | 4.67E-06 | 0.0344 |
| Synm | Protein coding | 1.6028 | 1.22E-05 | 0.0344 |
| Arrdc3 | Protein coding | -1.7612 | 1.29E-05 | 0.0344 |
| Jaml | Protein coding | 2.5400 | 1.55E-05 | 0.0352 |
| Gm8941 | Processed pseudogene | 1.3736 | 2.03E-05 | 0.0369 |
| Gm14097 | lncRNA | 1.9045 | 4.63E-05 | 0.0569 |
| Nrg4 | Protein coding | 1.7313 | 5.14E-0 | 0.0586 |

| | | | | |
|---|---|---|---|---|
| C730036E19Rik | lncRNA | 1.3406 | 5.88E-05 | 0.0626 |
| | | **Medium PAM Diet vs. Low PAM Diet** | | |
| **BC049987** | lncRNA | 1.8426 | 3.24E-07 | 0.0025 |
| **Mroh6** | Protein coding | 2.2508 | 3.3E-06 | 0.0102 |
| **Spr-ps1** | Transcribed unprocessed pseudogene | -2.0684 | 8.62E-06 | 0.0147 |
| **Agap2** | Protein coding | 1.2878 | 9.06E-06 | 0.0147 |
| **Mfsd2a** | Protein coding | 1.2067 | 1.72E-05 | 0.0187 |
| **Jun** | Protein coding | 1.3876 | 2.43E-05 | 0.0193 |
| **P4ha1** | Protein coding | 1.2183 | 2.49E-05 | 0.0193 |
| **Esrrb** | Protein coding | 2.0499 | 2.5E-05 | 0.0193 |
| **Tat** | Protein coding | 1.3202 | 2.85E-05 | 0.0199 |
| **Pla2g4f** | Protein coding | 3.6640 | 2.88E-05 | 0.0199 |
| **Gm3953** | Processed pseudogene | 2.5932 | 9.95E-05 | 0.0496 |
| **Synm** | Protein coding | 1.4124 | 0.0001 | 0.0577 |
| **Ctcflos** | lncRNA | 1.2053 | 0.0002 | 0.0671 |
| **Hspa1a** | Protein coding | 3.0351 | 0.0002 | 0.0730 |
| **G730003C15Rik** | lncRNA | -1.3837 | 0.0002 | 0.0773 |
| **Rdh18-ps** | Transcribed unprocessed pseudogene | 1.5895 | 0.0002 | 0.0773 |
| **Hsph1** | Protein coding | 1.7437 | 0.0004 | 0.0950 |
| **Tmc7** | Protein coding | 1.5846 | 0.0004 | 0.0950 |
| **Hhipl1** | Protein coding | 1.3329 | 0.0004 | 0.0952 |
| **Treh** | Protein coding | 1.4922 | 0.0004 | 0.0966 |
| **Itga10** | Protein coding | 1.2207 | 0.0004 | 0.0970 |

n = 5 male mice per diet (biological replicates). The Wald test was used to identify differentially expressed genes between samples in R Studio, with an adjusted p-value of either 0.05 or 0.1 and log2 fold change was set to 1.2. PAM, palmitic acid.

**Supplemental Table 3.** Top Significantly Differentially Expressed Gene Pathways Contrasting High Palmitic Acid and Low Palmitic Acid Diet Groups by Male Pup Liver and Brain Gene Set Enrichment Analysis at Day 35

| Direction | Pathways | NES | nGenes | Adjusted P-Value |
|---|---|---|---|---|
| | **Liver** | | | |
| Up | Regulation of cholesterol metabolic process | 2.1171 | 30 | 0.0022 |
| Up | Regulation of cholesterol biosynthetic process | 2.0883 | 17 | 0.0014 |
| Up | Regulation of sterol biosynthetic process | 2.0883 | 17 | 0.0014 |
| Up | Transforming growth factor beta production | 2.0544 | 29 | 0.0022 |
| Up | Regulation of the force of heart contraction | 2.0341 | 19 | 0.0031 |
| Up | Positive regulation of microtubule polymerization or depolymerization | 2.0264 | 32 | 0.0029 |
| Up | Collagen metabolic process | 2.0245 | 72 | 0.0012 |
| Up | Extracellular matrix organization | 2.0157 | 194 | 0.0011 |
| Up | Extracellular structure organization | 2.0157 | 194 | 0.0011 |
| Up | External encapsulating structure organization | 2.0157 | 194 | 0.0011 |
| Up | Regulation of collagen metabolic process | 1.9977 | 35 | 0.0035 |
| Up | Collagen biosynthetic process | 1.994 | 40 | 0.0051 |
| Up | Regulation of transforming growth factor beta production | 1.9798 | 27 | 0.0042 |
| Up | Positive regulation of cholesterol metabolic process | 1.9744 | 12 | 0.0051 |
| Up | Positive regulation of microtubule polymerization | 1.972 | 29 | 0.0047 |
| Up | Regulation of collagen biosynthetic process | 1.966 | 32 | 0.0042 |
| Up | Negative regulation of muscle adaptation | 1.9528 | 11 | 0.0063 |
| Up | Cell-matrix adhesion | 1.9417 | 175 | 0.0011 |
| Up | Positive regulation of cholesterol biosynthetic process | 1.9363 | 10 | 0.0069 |
| Up | Positive regulation of sterol biosynthetic process | 1.9363 | 10 | 0.0069 |
| Up | Negative regulation of cell-matrix adhesion | 1.9306 | 28 | 0.01 |
| Up | Muscle contraction | 1.9255 | 167 | 0.0011 |
| Up | Import across plasma membrane | 1.9084 | 98 | 0.0012 |
| Up | Regulation of cellular response to transforming growth factor beta stimulus | 1.9011 | 94 | 0.0012 |
| Down | Negative regulation of oligodendrocyte differentiation | -2.0123 | 11 | 0.01 |
| Down | Negative regulation of glial cell differentiation | -1.9662 | 19 | 0.021 |
| Down | Double-strand break repair via break-induced replication | -1.9416 | 12 | 0.024 |
| Down | Acyl-CoA biosynthetic process | -1.9287 | 29 | 0.021 |

| | | | | |
|---|---|---|---|---|
| Down | Thioester biosynthetic process | -1.9287 | 29 | 0.021 |
| Down | DNA strand elongation involved in DNA replication | -1.9087 | 10 | 0.029 |
| | **Brain** | | | |
| Up | Axoneme assembly | 1.9461 | 76 | 0.071 |
| Up | Cilium movement | 1.833 | 147 | 0.071 |
| Up | Feeding behavior | 1.8107 | 103 | 0.095 |
| Up | Microtubule bundle formation | 1.7818 | 109 | 0.095 |
| Up | Circadian rhythm | 1.7448 | 199 | 0.071 |
| Up | Reproductive process | 1.3571 | 1208 | 0.071 |
| Up | Reproduction | 1.3567 | 1209 | 0.071 |
| Down | Glutamate receptor signaling pathway | -1.953 | 54 | 0.095 |
| Down | Biological adhesion | -1.3719 | 1149 | 0.092 |
| Down | Cell adhesion | -1.3606 | 1139 | 0.092 |

n = 5 mice per diet (biological replicates). False discovery rate was set to 0.2. NES, normalized enrichment score; nGenes, number of genes.

**Supplemental Table 4.** Top Significantly Differentially Expressed Gene Pathways Contrasting Medium Palmitic Acid and Low Palmitic Acid Diet Groups by Male Liver and Brain Gene Set Enrichment Analysis at Day 35

| Direction | Pathways | NES | nGenes | Adjusted P-Value |
|---|---|---|---|---|
| | **Liver** | | | |
| Up | Mitotic sister chromatid segregation | 2.3743 | 145 | 0.0015 |
| Up | Sister chromatid segregation | 2.3091 | 170 | 0.0015 |
| Up | Mitotic cell cycle checkpoint signaling | 2.2969 | 104 | 0.0015 |
| Up | Regulation of mitotic metaphase/anaphase transition | 2.2943 | 53 | 0.0015 |
| Up | Metaphase/anaphase transition of mitotic cell cycle | 2.2899 | 54 | 0.0015 |
| Up | Mitotic sister chromatid separation | 2.2602 | 58 | 0.0015 |
| Up | Regulation of mitotic sister chromatid separation | 2.255 | 55 | 0.0015 |
| Up | DNA-dependent DNA replication | 2.244 | 125 | 0.0015 |
| Up | Mitotic spindle assembly checkpoint signaling | 2.2312 | 34 | 0.0015 |
| Up | Spindle assembly checkpoint signaling | 2.2312 | 34 | 0.0015 |
| Up | Mitotic spindle checkpoint signaling | 2.2312 | 34 | 0.0015 |
| Up | Negative regulation of mitotic cell cycle phase transition | 2.2305 | 124 | 0.0015 |
| Up | Negative regulation of mitotic metaphase/anaphase transition | 2.2217 | 36 | 0.0015 |
| Up | Spindle checkpoint signaling | 2.2194 | 35 | 0.0015 |
| Up | Regulation of metaphase/anaphase transition of cell cycle | 2.2149 | 55 | 0.0015 |
| Up | Negative regulation of metaphase/anaphase transition of cell cycle | 2.2106 | 37 | 0.0015 |
| Up | Metaphase/anaphase transition of cell cycle | 2.2097 | 56 | 0.0015 |
| Up | Chromosome separation | 2.2077 | 83 | 0.0015 |
| Up | Regulation of sister chromatid segregation | 2.2073 | 61 | 0.0015 |
| Up | DNA replication | 2.1992 | 224 | 0.0015 |
| Up | Mitotic nuclear division | 2.1981 | 241 | 0.0015 |
| Up | Cell cycle checkpoint signaling | 2.1823 | 137 | 0.0015 |
| Up | Negative regulation of mitotic nuclear division | 2.1812 | 44 | 0.0015 |
| Up | Nuclear chromosome segregation | 2.1773 | 223 | 0.0015 |
| Up | Negative regulation of sister chromatid segregation | 2.1767 | 37 | 0.0015 |
| Up | Negative regulation of mitotic sister chromatid segregation | 2.1767 | 37 | 0.0015 |
| Up | Negative regulation of mitotic sister chromatid separation | 2.1767 | 37 | 0.0015 |
| Up | Negative regulation of nuclear division | 2.1746 | 47 | 0.0015 |

| | | | | |
|---|---|---|---|---|
| Up | Negative regulation of chromosome segregation | 2.1663 | 38 | 0.0015 |
| Up | Negative regulation of chromosome separation | 2.1663 | 38 | 0.0015 |
| | **Brain** | | | |
| Up | Ammonium ion metabolic process | 2.011 | 24 | 0.12 |
| Up | Grooming behavior | 1.9868 | 18 | 0.14 |
| Up | Primary amino compound metabolic process | 1.9729 | 17 | 0.15 |
| Up | Regulation of bone remodeling | 1.9576 | 45 | 0.13 |
| Up | Regulation of bone resorption | 1.9541 | 37 | 0.15 |
| Up | Circadian rhythm | 1.9419 | 199 | 0.054 |
| Up | Serotonin metabolic process | 1.9297 | 15 | 0.18 |
| Up | Hyperosmotic response | 1.9275 | 28 | 0.18 |
| Up | Chaperone-mediated protein folding | 1.9269 | 56 | 0.12 |
| Up | Positive regulation of bone resorption | 1.9261 | 19 | 0.18 |
| Up | Chaperone cofactor-dependent protein refolding | 1.919 | 31 | 0.18 |
| Up | Epithelial cell maturation | 1.9157 | 16 | 0.18 |
| Up | Copulation | 1.9132 | 20 | 0.19 |
| Up | de novo posttranslational protein folding | 1.9118 | 35 | 0.18 |
| Up | de novo protein folding | 1.9115 | 36 | 0.18 |
| Up | Cellular response to interferon-alpha | 1.8954 | 13 | 0.18 |
| Up | Negative regulation of protein tyrosine kinase activity | 1.8918 | 28 | 0.2 |
| Up | Regulation of tissue remodeling | 1.8787 | 59 | 0.14 |
| Up | Bone remodeling | 1.8591 | 84 | 0.11 |
| Up | Maintenance of organelle location | 1.859 | 11 | 0.18 |
| Up | Neuron maturation | 1.8453 | 52 | 0.18 |
| Up | High-density lipoprotein particle remodeling | 1.831 | 9 | 0.18 |
| Up | Bone resorption | 1.7983 | 59 | 0.2 |
| Up | Embryonic skeletal system development | 1.7869 | 116 | 0.11 |
| Up | Regionalization | 1.7851 | 291 | 0.054 |
| Up | Cilium movement | 1.7694 | 148 | 0.078 |
| Up | Pattern specification process | 1.7651 | 376 | 0.054 |
| Down | Gas transport | -1.9007 | 13 | 0.078 |
| Down | Arachidonic acid metabolic process | -1.8108 | 27 | 0.19 |
| Down | Ectopic germ cell programmed cell death | -1.8021 | 7 | 0.078 |

n = 5 mice per diet (biological replicates). False discovery rate was set to 0.2. NES, normalized enrichment score; nGenes, number of genes.

**Supplemental Table 5.** Top 10 Enriched Pathways Among Genes in the Blue and Green Module by Liver Weighted Gene Co-Expression Network Analysis in Male Pups at Day 35

| Number of genes | Pathway Name | Accession | Adjusted P-value |
|---|---|---|---|
| | **Blue Module** | | |
| 50 | Lipid metabolic process | GO:0006629 | 4.9e-21 |
| 19 | Unsaturated fatty acid metabolic process | GO:0033559 | 2.8e-17 |
| 17 | Icosanoid metabolic process | GO:0006690 | 1.1e-14 |
| 14 | Arachidonic acid metabolic process | GO:0019369 | 1.1e-14 |
| 16 | Long-chain fatty acid metabolic process | GO:0001676 | 1.7e-14 |
| 45 | Small molecule metabolic process | GO:0044281 | 1.8e-13 |
| 24 | Fatty acid metabolic process | GO:0006631 | 1.8e-13 |
| 27 | Monocarboxylic acid metabolic process | GO:0032787 | 1.3e-12 |
| 16 | Olefinic compound metabolic process | GO:0120254 | 1.7e-12 |
| 13 | Response to xenobiotic stimulus | GO:0009410 | 1.9e-10 |
| | **Green Module** | | |
| 3 | Positive regulation of cell proliferation involved in kidney development | GO:1901724 | 9.9e-4 |
| 5 | Skeletal muscle cell differentiation | GO:0035914 | 9.9e-4 |
| 7 | Fat cell differentiation | GO:0045444 | 1.3e-3 |
| 6 | Skeletal muscle tissue development | GO:0007519 | 1.6e-3 |
| 4 | Circadian regulation of gene expression | GO:0032922 | 2.7e-3 |
| 3 | Cell proliferation involved in kidney development | GO:0072111 | 2.7e-3 |
| 5 | Regulation of fat cell differentiation | GO:0045598 | 3.7e-3 |
| 2 | Positive regulation of skeletal muscle cell differentiation | GO:2001016 | 5.6e-3 |
| 23 | Positive regulation of metabolic process | GO:0009893 | 5.6e-3 |
| 15 | Homeostatic process | GO:0042592 | 5.9e-3 |

n = 5 mice per diet. Soft threshold was set to 6 and a minimum module size of 20 was used. GO, gene ontology.

**Supplemental Table 6.** Pup Litter Size and Representation

| Birth date of litter: | Dam Identifier: | Cage Identifier: | Diet: | Total Litter Size (N): | Male (n): | Female (n): | Infanticide | P0 Male | P10 Male | P21 Male | P35 Male | P0 Female | P10 Female | P21 Female | P35 Female |
|---|---|---|---|---|---|---|---|---|---|---|---|---|---|---|---|
| **08/12/2019** | 901 | 1 | LP | 6 | 3 | 3 | | | | | X○ | | | | |
| **08/05/2019** | 903▽ | 2 | LP | 6 | 3 | 3 | | X | X | X | | | | | |
| **08/01/2019** | 904▽ | 3 | LP | 9 | 6 | 3 | | X | | X | X○ | X | X | X | |
| **07/30/2019** | 905▽ | 4 | LP | 8 | 5 | 3 | | X | X | X | X○ | X | X | X | |
| **08/01/2019** | 906▽ | 5 | LP | 5 | 3 | 2 | | X | | X | | | | | X |
| **08/01/2019** | 907▽ | 6 | LP | 6 | 3 | 3 | | X | X | X | | | | | X |
| **07/31/2019** | 908▽ | 7 | LP | 4 | 2 | 2 | | X | | | X○ | | X | X | |
| **08/01/2019** | 910▽ | 8 | LP | 7 | 4 | 3 | | X | X | X | X | | | | |
| **08/03/2019** | 913▽ | 10 | LP | 6 | 2 | 3 | | X | | | | | | | |
| **08/09/2019** | 914 | 11 | LP | 9 | 7 | 2 | | | | X | X○ | | | | |
| **08/03/2019** | 916▽ | 12 | MP | 7 | 5 | 2 | | X | X | X | X○ | | | | X |
| **07/31/2019** | 918▽ | 13 | MP | 6 | 3 | 2 | | X | | X | | X | X | | |
| **07/31/2019** | 919▽ | 14 | MP | 7 | 4 | 3 | | X | X | | X○ | | X | X | |
| **08/01/2019** | 921▽ | 15 | MP | 7 | 4 | 3 | | X | X | X | X○ | | | X | |
| **07/31/2019** | 922 | 16 | MP | 3 | 2 | 1 | X | | | | | | | | |
| **08/03/2019** | 924▽ | 18 | MP | 3 | 3 | | | X | | X | X○ | | | | |
| **08/01/2019** | 925▽ | 19 | MP | 4 | 3 | 1 | | | X | X | | | | X | |
| **08/11/2019** | 926 | 20 | MP | 8 | 5 | 3 | | | X | | X○ | | | | |
| **07/30/2019** | 927▽ | 21 | MP | 6 | 3 | 3 | | | | X | | X | | | |
| **08/04/2019** | 928▽ | 22 | HP | 6 | 5 | 1 | | X | X | X | X○ | | | | |
| **08/02/2019** | 931▽ | 24 | HP | 2 | 2 | | | X | | | X | | | | |
| **08/05/2019** | 932▽ | 25 | HP | 5 | 2 | 3 | | X | X | | | | X | | |
| **08/04/2019** | 933▽ | 26 | HP | 7 | 5 | 2 | | X | X | X | X○ | | X | | |
| **08/09/2019** | 934 | 27 | HP | 7 | 4 | 3 | | | | X | | | | | |
| **07/31/2019** | 935▽ | 28 | HP | 8 | 6 | 2 | | X | X | X | X○ | X | | | |
| **08/11/2019** | 936 | 29 | HP | 9 | 6 | 3 | X | | | | | | | | |

| | | | | | | | | | | | | | | | |
|---|---|---|---|---|---|---|---|---|---|---|---|---|---|---|---|
| **07/30/2019** | 937▽ | 30 | HP | 9 | 5 | 4 | | X | X | X | X○ | X | | | X |
| **08/01/2019** | 938▽ | 31 | HP | 7 | 4 | 3 | | X | X | X | X○ | X | | X | |
| **08/05/2019** | 939▽ | 32 | HP | 5 | 3 | 2 | | X | X | X | | | | | X |

HP, high palmitic acid; LP, low palmitic acid; MP, medium palmitic acid; P0, postnatal day 0; P10, postnatal day 10; P21, postnatal day 21; P35, postnatal day 35.

[X]Primary outcome data (brain and liver $\delta^{13}$C fatty acid values, brain, and liver fatty acid concentration/relative percentage)

◦RNAseq

[▽]Litter underwent behavioural testing
