# Supplementary material for: Upregulated hepatic lipogenesis from dietary sugars in response to low palmitate feeding supplies brain palmitate
Source: Nat Commun. 2024 Jan 17;15:490. doi: 10.1038/s41467-023-44388-4 (PMC10794264; doi:10.1038/s41467-023-44388-4)
Supplement: Supplementary file 3 — Reporting Summary [file 41467_2023_44388_MOESM3_ESM.pdf]

nature portfolio

Corresponding author(s): Dr. Richard P Bazinet

Last updated by author(s): Nov 11 2023

Reporting Summary

Nature Portfolio wishes to improve the reproducibility of the work that we publish. This form provides structure for consistency and transparency in reporting. For further information on Nature Portfolio policies, see our Editorial Policies and the Editorial Policy Checklist.

Statistics

For all statistical analyses, confirm that the following items are present in the figure legend, table legend, main text, or Methods section.

n/a	Confirmed	
☐	☒	The exact sample size (*n*) for each experimental group/condition, given as a discrete number and unit of measurement
☐	☒	A statement on whether measurements were taken from distinct samples or whether the same sample was measured repeatedly
☐	☒	The statistical test(s) used AND whether they are one- or two-sided<br>*Only common tests should be described solely by name; describe more complex techniques in the Methods section.*
☒	☐	A description of all covariates tested
☐	☒	A description of any assumptions or corrections, such as tests of normality and adjustment for multiple comparisons
☐	☒	A full description of the statistical parameters including central tendency (e.g. means) or other basic estimates (e.g. regression coefficient) AND variation (e.g. standard deviation) or associated estimates of uncertainty (e.g. confidence intervals)
☐	☒	For null hypothesis testing, the test statistic (e.g. *F*, *t*, *r*) with confidence intervals, effect sizes, degrees of freedom and *P* value noted<br>*Give P values as exact values whenever suitable.*
☒	☐	For Bayesian analysis, information on the choice of priors and Markov chain Monte Carlo settings
☒	☐	For hierarchical and complex designs, identification of the appropriate level for tests and full reporting of outcomes
☒	☐	Estimates of effect sizes (e.g. Cohen's *d*, Pearson's *r*), indicating how they were calculated

Our web collection on statistics for biologists contains articles on many of the points above.

Software and code

Policy information about availability of computer code

Data collection	No code was used in this study to collect data, code was only used for data analysis which has been reposited in Zenodo and described in the Data anlysis section below.
Data analysis	R code for results obtained in iDEP.96 (GSEA and WGCNA) are publicly available (https://idepsite.wordpress.com/degs/). R code used for PCA analysis, DEG analysis as well as volcano plot construction have been deposited in Zenodo under DOI: DOI: 10.5281/zenodo.10137505 (which has been added to the code availability section of the manuscript). All softwares used with versions (iDEP .96, R 4.2.1., Isodat 3.0, CompassCDS 3.0) have been mentioned in the manuscript.

For manuscripts utilizing custom algorithms or software that are central to the research but not yet described in published literature, software must be made available to editors and reviewers. We strongly encourage code deposition in a community repository (e.g. GitHub). See the Nature Portfolio guidelines for submitting code & software for further information.

Data

Policy information about availability of data

All manuscripts must include a data availability statement. This statement should provide the following information, where applicable:

- Accession codes, unique identifiers, or web links for publicly available datasets
- A description of any restrictions on data availability
- For clinical datasets or third party data, please ensure that the statement adheres to our policy

The RNA-seq data generated in this study have been reposited in the NCBI Gene Expression Omnibus (GEO) database under accession code GSE225568 [https://www.ncbi.nlm.nih.gov/geo/query/acc.cgi?acc=GSE225568] (The accession code that was private, has been made public as of November 10th, 2023). All other main text and supplemental data generated in this manuscript are provided in the source data file. Additionally, mass-spectrometry files (.dxf) used in generating primary outcome data - δ13C-PAM brain and liver values; has been deposited in Zenodo and are publicly accessible under DOIs: 10.5281/zenodo.10139336 and 10.5281/zenodo.10140476.

Research involving human participants, their data, or biological material

Policy information about studies with human participants or human data. See also policy information about sex, gender (identity/presentation), and sexual orientation and race, ethnicity and racism.

Reporting on sex and gender	N/A
Reporting on race, ethnicity, or other socially relevant groupings	N/A
Population characteristics	N/A
Recruitment	N/A
Ethics oversight	N/A

Note that full information on the approval of the study protocol must also be provided in the manuscript.

Field-specific reporting

Please select the one below that is the best fit for your research. If you are not sure, read the appropriate sections before making your selection.

☒ Life sciences ☐ Behavioural & social sciences ☐ Ecological, evolutionary & environmental sciences

For a reference copy of the document with all sections, see nature.com/documents/nr-reporting-summary-flat.pdf

Life sciences study design

All studies must disclose on these points even when the disclosure is negative.

Sample size	No sample size calculation was performed as investigating mouse brain PAM origin during development in response to our custom diets has not yet been explored. Nevertheless, our sample size was chosen based on a similar studies from our group investigating brain fatty acid δ13C-values in response to diet using n = 3-4 mice per group (https://www.ncbi.nlm.nih.gov/pmc/articles/PMC10154972/). Because our technique, GC-C-IRMS, is very precise and is capable of detecting standard deviations up to 4-6 significant figures as well as sensitive below 0.001 atom % excess, a smaller number of mice per group is sufficient to detect significant differences between groups.
Data exclusions	Exclusion criteria for mice throughout the study was clearly defined a priori. No pups were excluded throughout the study according to defined exclusion criteria. However, 2 litters were excluded from the study due to dam infanticide. Individual data for outcomes was only excluded when formally identified as an outlier using the ROUT method in Graph Pad Prism Version 9 - which is reported in Supplemental Figure 2E and F in the methods section and source data file.
Replication	Significant efforts were taken to ensure reproducibility of our work including the use and mention of authentic internal (ex. unesterified 17:0) and reference standards (ex. GLC-569) within the methods section, in addition to all reagent catalogue numbers, diet composition catalogue numbers, information on mouse strain, housing conditions, etc.
Randomization	Randomization of the dams to each of the low, medium and high PAM diets was performed using simple randomization with stratification for body weight.
Blinding	Blinding was not possible in our study as feeding of the study diets, behavioral tests, lab analyses, and statistical analyses were performed by one investigator (MES).

Reporting for specific materials, systems and methods

We require information from authors about some types of materials, experimental systems and methods used in many studies. Here, indicate whether each material, system or method listed is relevant to your study. If you are not sure if a list item applies to your research, read the appropriate section before selecting a response.

Materials & experimental systems

n/a	Involved in the study
☒	☐ Antibodies
☒	☐ Eukaryotic cell lines
☒	☐ Palaeontology and archaeology
☐	☒ Animals and other organisms
☒	☐ Clinical data
☒	☐ Dual use research of concern
☒	☐ Plants

Methods

n/a	Involved in the study
☒	☐ ChIP-seq
☒	☐ Flow cytometry
☒	☐ MRI-based neuroimaging

Animals and other research organisms

Policy information about studies involving animals; ARRIVE guidelines recommended for reporting animal research, and Sex and Gender in Research

Laboratory animals	BALB/c female mice - ordered at 32 weeks of age (Saint Constant, QC, CA; RRID: IMSR_CRL:028) were housed (n = 4 per cage) within the Division of Comparative Medicine at the University of Toronto which maintained a constant light cycle (12 h light/12 h dark), controlled temperature (21°C), and 40-60% humidity throughout the study. Housing through breeding and pre- and post- weaning maintained the same conditions, and pups per cage is reported throughout the manuscript.
Wild animals	Our study did not involve wild animals.
Reporting on sex	Unfortunately, all dams in our study produced a significantly higher number of male pups, to female pups ($p < 0.0001$; Supplemental Figure 13) in their litters. Therefore, the primary outcomes in our study (δ13C data and RNAseq) are powered to assess male pups (n = 4-8 male pups) and a subset (n = 1-3) of female pups were taken, when possible, to assess if females responded in a similar manner to our study diets. However, sample size for female pups was too small to conduct any statistical analyses. Therefore, the sex-bias in our study for males has been reported in the title and the results.
Field-collected samples	Our study did not involve samples collected from the field.
Ethics oversight	The animal use protocol (20012290), conducted in accordance with the Canadian Council on Animal care, was approved by the animal ethics committee at the University of Toronto and is referenced to in the manuscript.

Note that full information on the approval of the study protocol must also be provided in the manuscript.
